# Integrated Education Models for Addressing Substance Use Among Schoolchildren: A Scoping Review of School-Based Educational Interventions

**DOI:** 10.3390/ijerph23081073

**Published:** 2026-08-18

**Authors:** Deeksha Bajpai Tewari, Upma Gautam, Vaishnavi Jaiswal, Pooja, Ankita Mishra, Priya Das

**Affiliations:** 1 Department of Geography, Dyal Singh College, University of Delhi, New Delhi 110003, India; deekshabajpai@dsc.du.ac.in; 2 University School of Law & Legal Studies, Guru Gobind Singh Indraprastha University, Dwarka, New Delhi 110078, India; vaishnavi.12jaiswal@gmail.com (V.J.); ankitamadv@gmail.com (A.M.); 3 University School of Education, Guru Gobind Singh Indraprastha University, Dwarka, New Delhi 110078, India; pooja1984@ipu.ac.in; 4 Campus Law Centre, Faculty of Law, University of Delhi, New Delhi 110007, India; priyadas@clc.du.ac.in

**Keywords:** educational interventions, drug use, prevention, protection, correction, school students

## Abstract

**Highlights:**

**Public health relevance—How does this work relate to a public health issue?**

- Substance use among school-going children remains a significant global public health concern, with an estimated 37 million adolescents aged 13–15 years using tobacco and over 50% of 15-year-olds reporting alcohol use worldwide.
- Early substance uses increases risk of substance use disorders, unsafe sexual behaviour, and drug-related injury, posing serious physical and mental health risks.

**Public health significance—Why is this work of significance to public health?**

- This scoping review synthesises evidence from 43 studies and 33 distinct intervention models, addressing a gap in the literature by thoroughly mapping the design features that make school-based substance use prevention effective.
- The findings show that interventions grounded in explicit theory and involving multiple stakeholders (parents, community, and experts) produce more consistent and sustained reduction in substance use.

**Public health implications—What are the key implications or messages for practitioners, policy makers and/or researchers in public health?**

- Practitioners and policymakers should design interventions combining theoretical grounding, multi-stakeholder involvement, and dosage calibrated to children’s developmental stage, rather than relying on content volume alone.
- No corrective intervention model currently exists for schoolchildren already using substances. Researchers and policymakers should prioritise developing non-stigmatising, therapeutic school-based alternatives to punitive responses.

**Abstract:**

Background: Early and comprehensive interventions are crucial for preventing substance use in adolescents. In school settings, such interventions can be easily integrated into the curricular and co-curricular activities with the active involvement of school administration, teachers, trained staff, parents, and community members. This review aimed to comprehensively examine preventive, protective, and corrective school-based intervention models for substance use to suggest an evidence-based and effective educational model for preventing substance use in children. Methods: In this scoping review, online databases, including SCOPUS and Web of Science, were searched to identify and collect articles evaluating the effectiveness of school-based prevention models. The PRISMA approach for scoping review (PRISMA-ScR) was employed to assess the scope of relevant literature and synthesise existing school-based intervention models. Two independent reviewers independently screened records (κ = 0.75). Due to heterogeneity, findings were synthesised narratively using a socio-ecological framework. Study quality was appraised using the Mixed Methods Appraisal Tool (MMAT). Results: Forty-three studies, mostly from the United States, were included in the review. All school-based interventions examined were preventive or protective in their approaches. There was no school-based intervention model that was corrective in its approach. Increased knowledge, negative attitudes towards substance use, and reduced substance use in the intervention groups were key outcomes. Effective interventions involved a multi-pronged approach wherein teacher training, student education, and the involvement of parents, senior citizens, police officials, experts, and the community were key components. Additionally, culturally tailored interventions have shown promising results. Conclusions: Among school children, tobacco and alcohol use are most prevalent, and schools can play a crucial role in addressing this issue. Apart from preventive and protective intervention models, the focus needs to shift towards developing integrated school-based corrective models to address the concerns of substance use among school students.

## 1. Introduction

Early substance use threatens physical, mental, and social well-being, risking substance use disorders (SUDs) [1,2,3,4,5,6]. Alcohol, tobacco, and illicit drugs, especially cannabis, are commonly used substances by young adolescents in their early stages of life [7,8]. Globally, around 37 million youth aged about 13 to 15 years use tobacco in some form [9]. Another WHO survey demonstrated that the use of alcohol among 15-year-olds was even more widespread (>50%) [10]. Furthermore, the World Drug Report 2025 found that use of illicit drugs like amphetamines, cocaine and ecstasy is higher among the population aged 15–16 years than the general population [11].

Individual risk factors among adolescents include peer pressure [12,13], experimentation [14,15], boredom and curiosity [16], misinformation [17], misguided coping mechanisms [8,18], and impulsive decision-making [19,20]. Environmental drivers, like exposure to substance use within families [21,22], financial hardships, stressed neighbourhoods [23], and easy access to substances [24,25], further drive the early onset of substance use among school children [26,27]. Early onset of drug use among adolescents further increases the risk of maladaptive behaviours: poor academic performance [28], absenteeism [29], unsafe sexual activities [30,31], STDs [32], and delinquency [33].

Intervention strategies reduce these risk factors more efficiently when it is initiated at an early age, targeting specific risk factors [34,35]. Studies have developed various strategies, including parent-focused skill building, interactive school programmes, targeted high-risk programmes, multi-level prevention strategies, and trauma-focused prevention strategies [36,37,38,39].

School-based intervention models are the most effective approach to reach a large early-age population with substance use awareness, as they provide routine access to huge numbers of children during the risk period when initiation often begins [40].

Furthermore, school education aids in preventing substance use among children and reshaping their behaviour [41]. Inclusive school-based preventive intervention models significantly reduce substance use risk [42]. These interventions draw on theoretical frameworks addressing the risk and protective factors [43,44], family-based [45], youth empowerment [46], cognitive and behavioural, community and social learning approaches [47,48].

Family-integrated school interventions address risk factors such as poor parental monitoring and family conflict [39,49,50]. Furthermore, integrating senior citizens, trained counsellors, civil society members, and state functionaries may form part of strategic preventive models for reducing substance use among young adolescents [51]. The composition of the models may vary depending on the specific age group, grade, race, ethnicity, and skills targeted by the intervention [52,53].

Preventive interventions for substance use introduced in school settings can influence children’s behaviour by including the components of knowledge impartation [54] and skill development through socialisation [55]. School settings uniquely enable broad access to children and easier identification of vulnerability, making them ideal for multi-dimensional interventions [56]. Moreover, such interventions should be early and structurally tailored against the backdrop of socio-cultural sensibilities [57] to address the specific needs and behavioural risk factors of school-going young cohorts [58].

The substance use literature illustrates that preventive interventions are not sufficient on their own and emphasises strengthening protective and corrective approaches once risk factors, harmful use or related problems appear [59,60]. Prior systematic reviews, meta-analyses, and individual trials offer a mixed picture of school-based substance use prevention effectiveness. A systematic review of 29 randomised controlled trials found that skills-based interventions significantly reduced marijuana use and hard drug use compared with usual curricula [61]. At the level of individual trials, randomised evaluation of Life Skills Training across 56 New York schools similarly found reduced tobacco, alcohol, and marijuana use among adolescents receiving the programme [62], with 13-year follow-up later showing that these reductions extended into reduced illicit drug use in young adulthood [63]. A meta-regression showed that programme effectiveness is not uniform across development, with specific intervention characteristics working for some age groups but not others [64]. Altogether, these studies suggest that skills-based, social competency-focused content, delivered with attention to developmental stage and evaluated independently rather than solely by programme developers, is most consistently linked to favourable outcomes, while generic or developer-evaluated-only curricula show weaker or less certain effects.

Despite this accumulating evidence, two gaps remain insufficiently addressed. First, existing reviews and individual trials largely evaluate programme components (e.g., skills training, developmental timing, independence of evaluation) in isolation, without examining how theoretical grounding, multi-stakeholder involvement, cultural adaptation, and dosage interact within the same intervention. Second, most reviews and trials focus on preventive interventions for substance-naïve populations, leaving protective and corrective approaches for students already showing risk or early use comparatively unmapped. This scoping review addresses these gaps by jointly mapping preventive, protective, and corrective school-based intervention models, examining how theoretical grounding, stakeholder involvement, cultural adaptation, and dosage/developmental timing interact to shape outcomes, and identifying where the evidence base remains thin.

For this review, preventive models are defined as those delivered to substance-naïve populations before any use has occurred; protective models as those that build resilience or resistance skills in general or identified at-risk populations without requiring prior use; and corrective models as those explicitly designed to intervene with students who have already initiated substance use.

The research objective is framed within the PCC framework below (Table 1).

### Research Questions

This review aims to answer the following questions:

- Do explicit theoretical underpinned intervention models of substance use produce better and more sustained outcomes than atheorised interventions?
- What is the impact of specific stakeholder combinations in school-based substance use interventions on behavioural outcomes?
- Which specific psychosocial skill sets most strongly predict reduced substance use initiation?

## 2. Material and Methods

The scoping review approach was utilised to collect research evidence, which aids researchers in establishing the extent of the relevant literature around a broad study area. This method is beneficial for understanding the conceptual boundaries of a topic. It also aids in providing evidence-based suggestions for new policies or reforms in existing policies. This study utilised the PRISMA 2020 for scoping review (PRISMA-ScR) to scope the assessment and synthesise the models and the Preferred Reporting Items for Systematic Reviews and Meta-Analyses extension for Scoping reviews checklist (PRISMA-ScR) [Supplementary Materials File S1]. No protocol was pre-registered for this scoping review. Additionally, the review utilises a narrative synthesis approach, consistent with advanced scoping review methodology [65].

### 2.1. Search Strategy

This study comprises the relevant literature extracted from online databases, SCOPUS and Web of Science (refer to Table 2). Both databases were searched from inception to December 2025, the date of the original review’s final search. As part of this revision, the search was re-run on 3 August 2026, using the broadened search string described below; the coverage end-date was not extended beyond December 2025. This deliberately isolates the effect of the broadened search terms from that of the newly published literature, so that any additional records identified can be attributed to the revised search string rather than to the passage of time. The search string, provided in Table 2, was used within the data fields of title, abstract, keyword plus, and author keywords. Relative to the original review, a few new terms were added in the existing blocks, namely “Alcohol”, “Vaping”, “e-cigarette”, “Cigarette”, “Adolescent”, “Youth”, “Students”, “Young”, “Project”, and “RCT”, while the concept block (“Attitude” OR “Practices” OR “Knowledge”) was removed entirely to widen the search.

### 2.2. Eligibility Criteria

Studies included in this review fulfilled the following inclusion criteria: (1) no time limits as to the date of publication of studies; (2) available in English; (3) full access; (4) evaluated the efficacy of school-based preventive/protective/corrective interventions against substance use through quantitative, qualitative, or mixed methods; and (5) must have been conducted in school settings. The exclusion criteria were as follows: (1) book chapters, conference papers, and dissertations; (2) articles without full text and abstract; (3) studies that did not include any other intervention model other than substance use/abuse (like mental health, suicide, obesity) and (4) studies conducted on school students but without any involvement of the school administration (e.g., purely community-based or purely family-based interventions).

### 2.3. Data Charting

Two reviewers independently screened titles and abstracts, achieving an inter-rater reliability of κ = 0.75, indicating substantial agreement [66]. Discrepancies were resolved through discussion with a third reviewer. Thereafter, full texts of the remaining articles were read to determine the variables that could be extracted for this study. After understanding the intervention models provided in the study, they made a joint decision to chart the data graph based on the selected data items. Quality appraisal used the Mixed Methods Appraisal Tool (MMAT), which evaluates studies against design-specific criteria across quantitative, qualitative, and mixed-methods designs. Quality was not used as an exclusion criterion, consistent with scoping review methodology, but informed the interpretation of findings.

### 2.4. Data Items

The following details were extracted from the selected studies: name of intervention, country in which the study was conducted, theory on which the intervention was based, study design (including method, sample, and design employed for performing the analysis, duration), age or grade of the school students kept as the targeted population in the study, participants of the intervention, composition of the intervention, skills targeted in the study, and outcome of the study.

## 3. Results

**Figure 1:**
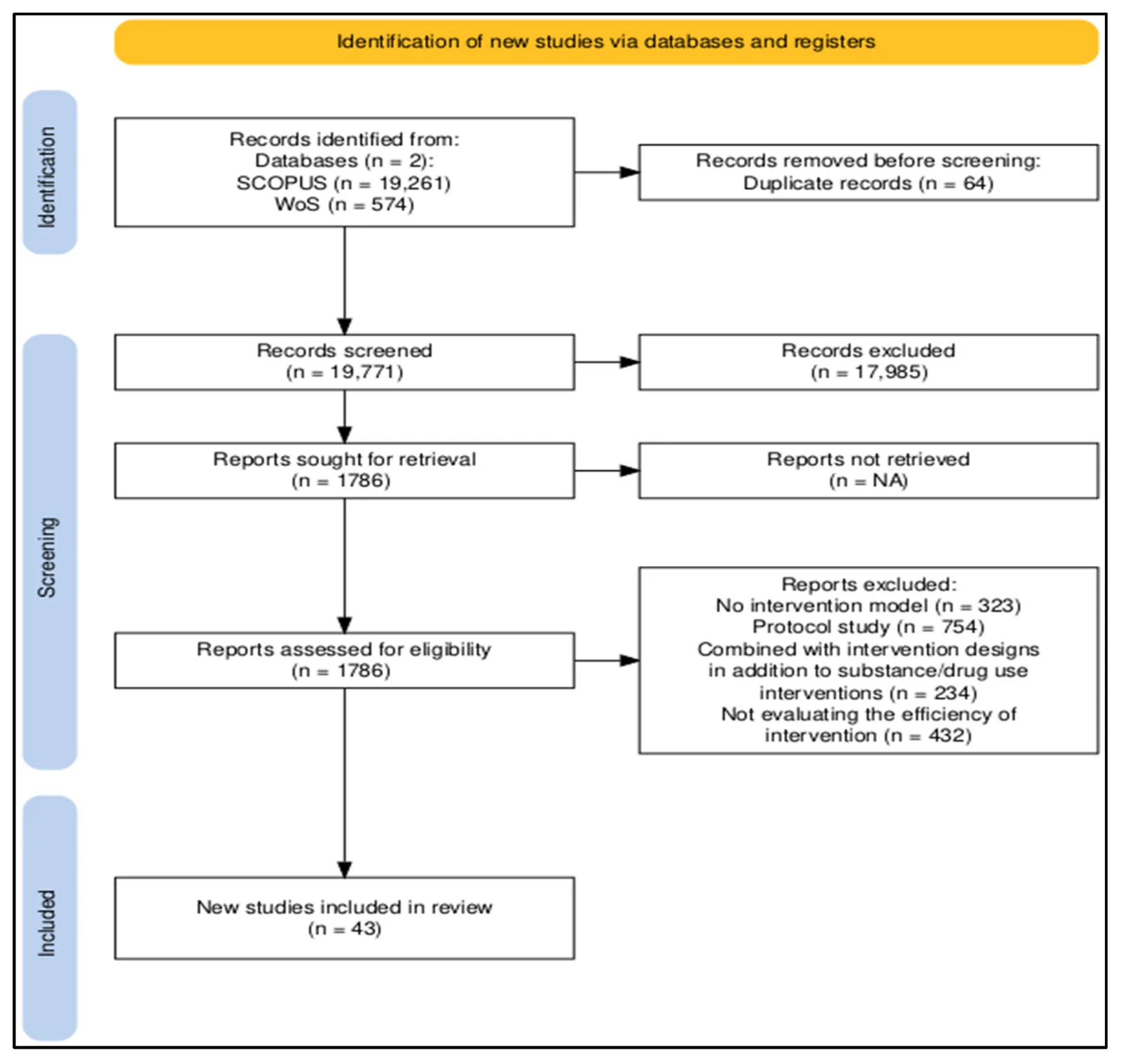
PRISMA flow diagram for scoping reviews.

Of the 19,835 studies extracted from the two databases, 64 among them were extracted as duplicates and thereafter 17,985 records were excluded after title and abstract screening as they do not meet the eligibility criteria. From the remaining 1786 studies, (i) 323 were excluded as they did not include any specific school-based substance use intervention model; (ii) 754 were the protocol study; (iii) 234 studies included combined approaches wherein they analysed other types of interventions along with the substance use intervention model; (iv) 432 articles provided a blueprint of the intervention models and do not address or evaluate their practical efficiency. Ultimately, only 43 studies met all the inclusion criteria and were included in this scoping review (Figure 1).

### 3.1. Overview of Intervention Models

The intervention models in this review were either in their original form or modified with enhancements like acculturation, electronic or digital modes, and other alterations. Of forty-three, seven examined the efficacy of the keepin’ it R.E.A.L. (kiR) model [67,68,69,70,71,72,73]. The kiR model uses resistance strategies, (R)efusal, (E)xplain, (A)void, and (L)eave, based on the premise that most children who initiate substance use at an early age do so not out of the desire to try it, but because they lack the requisite social skills to resist drug offers. This model consists of multiple integrated elements to teach children knowledge, motivation and communication skills to enhance their capacity to refuse drug offers. These elements include 10 interactive classroom sessions, five videos scripted, acted and filmed by students and booster activities after every classroom session (Table 3). The Allgemeine Lebenskompetenzen und Fertigkeitan (ALF) model aims to enhance students’ general competence through life skills [74]. It emphasised protective factors rather than risk factors. It consists of two main parts: general competence enhancement sessions focusing on learning skills to manage everyday life challenges and substance-specific content including information about negative consequences of nicotine and alcohol use.

The D.A.R.E. project is a school-based primary prevention programme delivered by uniformed police officers in the United States, designed to prevent drug abuse among elementary school students during their transition to middle school [75]. The programme operates for 1 h per week over 17 consecutive weeks and integrates multiple theoretical approaches: an informational approach focusing on drug education and effects, an affective approach targeting self-esteem enhancement and emotional awareness, and a social influence approach emphasising peer pressure resistance and decision-making skills.

Take Charge of Your Life (TCYL) also includes D.A.R.E. as an element [76]. It is a universal, school-based substance abuse prevention programme designed to prevent the use of alcohol, tobacco, and marijuana among adolescents in the United States. The intervention comprises two core components: a 10-lesson curriculum delivered in 7th grade and a 7-lesson booster program in 9th grade, both addressing normative beliefs, consequences of substance use, decision-making, and resistance skills.

The Across Ages’ model pioneered an inclusive, cross-generational mentoring approach, involving older adults (55+) as mentors to high-risk students, and as recipients of support, to teach students skills like responsibility, decision-making, and providing support [77]. The model is designed around the mission to increase the resilience and protective factors among students across five domains: the family, the peer group, the community, the school, and the individual. This intervention includes four major elements: intergenerational mentoring, classroom-based life skills, curriculum and parental involvement.

The All-Stars intervention model [78] addresses four mediating factors: personal commitment against high-risk behaviours, principles incompatible with such behaviours, bonds with prosocial establishments, and conservative thought processes regarding high-risk behaviours. This intervention was initiated as smoking prevention research and now includes every major form of substance use, like tobacco, marijuana, inhalants, cocaine and alcohol. Interactive classroom, small group and individual sessions are the activities included in the model for systematically inculcating the four mediating factors in the students.

The CLIMATE Schools is a computerised school-based intervention with a harm minimization approach designed to decrease alcohol misuse among adolescents [79]. The intervention consisted of six 40 min lessons. Each lesson included two sections: the first is a 15–20 min computer-based lesson and the second consists of class activities. This blended approach enabled teachers to deliver standardised, accurate content through online material while facilitating interactive communication and translating the content into students’ own lives through classroom activities.

The Climate and Preventure (CAP) study combined two complementary prevention approaches [80]. The universal Climate Schools comprises twelve 40 min lessons. Each lesson integrates a 20 min online cartoon component completed individually by students, followed by a 20 min teacher-led class activity that reinforces content and enables interactive communication. The selective Preventure programme targets high-risk students identified on four personality subscales through two 90 min group sessions delivered one week apart by registered clinical psychologists. Sessions employ psycho-educational strategies and cognitive-behavioural approaches to educate students about personality-specific risk factors (negative thinking, anxiety sensitivity, impulsivity, or sensation seeking) and help them challenge problematic thought patterns.

The Creciendo Juntos (Growing up Together) model is a school prevention programme implemented since 2013 to address substance use among students through a comprehensive, community-wide approach [81]. The intervention’s delivery system involves trained MT (Madrid Tutor) coordinators and operators who facilitate semester-long group projects tailored to student interests and needs. To ensure programme integration across the school, parental engagement is facilitated through conferences, workshops, and a dedicated programme microsite.

The Coping Power Program, which is a multicomponent preventive intervention for aggressive children based on Social Cognitive, Social Learning Theory, and Developmental Theory. This model addresses potential future risks like delinquency and substance use by addressing both parent-level and child-level factors [82]. The two major components covered by the model are: firstly, the child component, a 1.25-year programme delivered in a school setting consisting of 33 weekly group sessions led by specialist staff (master’s or doctoral degree in psychology or social work) and a school guidance counsellor; secondly, the parent component, a 16-session training programme for parents teaching them skills of managing children and strengthening the family communication.

Another theoretically based model, Drug Education in Victorian Schools (DEVS), derives its principles from Social Learning Theory, post-structural subjectivity theory, and cognitive dissonance theory [83] and provides integrated education on licit and illicit drugs, specifically alcohol. It is a two-year harm minimisation-focused drug education initiative for junior secondary school students. It comprises 18 lessons delivered during a period of 2 years. Lessons are on the use of alcohol and other drugs and explore the broader issues of drug use, like violence, antisocial behaviour, mental health and sexual vulnerability.

The School Health and Alcohol Harm Reduction Project (SHAHRP), an Australian harm-minimisation model closely related in philosophy to DEVS, aimed to reduce alcohol-related harm by enhancing students’ ability to identify and manage high-risk drinking situations [84]. Delivered over two phases across a 32-month period, it comprised eight activity-based classroom lessons in the first year followed by five booster lessons in the second, with a teacher manual, student workbooks and videos supporting delivery.

The Drug Story Theatre (DST) uses a strategic prevention framework and peer-to-peer approach to inform middle and high school students about substance use risk. It reframes addiction and SUDs as a health problem and not a moral failing [85]. This model includes theatrical performances developed and enacted by students in early stages of their recovery, performed before middle and high school students in a school setting. It also includes brief presentations about adolescent brain development in between the scenes. Following the show, audiences engage in a talk-back session with performers moderated by a professional with expertise in adolescent substance use.

The Fresh Start model, based on the Theory of Planned Behaviour, the Health Belief Model (HBM), and the Transtheoretical Model, offers a school-based intervention programme for secondary school freshmen. It aids students to retain negative attitudes toward substance use during the transition period from primary to secondary school, a period during which abstract thinking develops [86]. The model consists of four weekly lessons of 50 min each, with a total duration structured to fit within the school year and also includes a parent conference on parenting early adolescents.

The Police, Public educators, and Peers Utilising Leadership skills of Students At Risk/As Resource (PULSAR) is designed as a qualitative, resiliency-focused intervention targeting students identified as at-risk [87]. It operates through several core mechanisms, including self-reflection facilitation, skills development, and relationship building. Students are provided with activities related to team building, group processes, self-assessment and self-awareness, group assessment, school, and community involvement.

The Substance Use Prevention Education Programme (SUPEP) intervention uses the HBM. Its contents integrate the U.S. and national health education resources [88]. It is a school-based intervention designed to address psychoactive substance use among young people. It focuses on enhancing knowledge, attitudes and behaviours related to substance use through evidence-based educational approaches. This intervention includes standardised interactive sessions for students once a week, teachers’ training using the WHO primary prevention of substance abuse training manual and facilitator guide, and the SUPEP manual to facilitate effective programme delivery.

The Healthy School and Drugs Project targets students aged 12–18 years. Based on the Theory of Planned Behaviour, the Social Cognitive Theory, and the Model of Behavioural Change, it utilises the ASE approach, which explains behaviour and intention through attitude (individual judgement of anticipated behaviour), social influence (opinions and expectations of others regarding that behaviour), and self-efficacy (personal confidence in achieving a specific behaviour) [89].

Another intervention based on Social Cognitive Theory is CATCH My Breath [90,91]. It is an evidence-based vaping prevention programme developed by researchers at the University of Texas School of Public Health, incorporating evidence from youth tobacco prevention programmes and risk-factors data. This program has age-specific curricula. Furthermore, it includes two components: four classroom sessions of 40 min delivered in-person or via virtual sessions and peer-led small group interactive activities focusing on the development of targeted skills (communication, self-esteem, and resistance against social pressure).

The OurFutures Vaping programme is a school-based eHealth intervention designed to prevent e-cigarette use among adolescent students in Australia [92]. It comprises four 40 min lessons about e-cigarettes and tobacco (including prevalence rates, associated harms, refusal strategies, and help-seeking), delivered approximately 1 week apart. Each lesson comprised a web-based cartoon with embedded quizzes and reflective activities.

The HeadOn Intervention is a self-guided, computer-based intervention designed for 6th to 8th graders to promote recognised “protective factors” against substance use, incorporating continuous feedback from middle school students during development [93]. This programme is designed on two key informational technologies: computer-based modules and interactive game components. The modules address key prevention topics such as consequences and risks of experimentation with drugs, drug-refusal skills training, self-management and social skills. Additionally, the “Skills Challenge” game is an electronic card game that enables students to gain practical knowledge on skills learned via modules. Teachers actively supervised the implementation of programmes in schools with an electronic tracking system to view detailed reports of students’ activities.

The Too Good For Drugs (TGFD) programme aims to protect youth from alcohol, tobacco, and other drug (ATOD) usage by enhancing protective factors and reducing associated risk factors through a social influence-based approach [94]. This is the only programme in the study that covers grades K to 12th. It employs highly interactive teaching sessions through small group activities, interactive games, role-playing activities, class discussion, and iterative practice. The programme covers lessons on goal setting, decision-making, managing emotions, effective communication, interpersonal bonding, and knowledge of ATOD and the consequences of its use.

Click City is an online, school-based curriculum for 5th graders with a 6th-grade booster, originally developed for cigarette and smokeless-tobacco prevention and later updated to also target e-cigarettes, drawing on the prototype/willingness model and the theories of planned behaviour and reasoned action [95]. Delivered individually by students on school computers under teacher supervision, the programme comprises eight 20–25 min lessons targeting favourable social images of smokers/vapers, normative beliefs, and perceived risks of health consequences and addiction.

Project ALERT also targets middle schoolers to prevent initiation or reduce substance initiation, namely cigarettes, alcohol, marijuana, and inhalants [96,97]. It is a 2-year, manualized substance use prevention curriculum. This intervention strategy included guided class discussions, small group activities, role-playing exercises, and videos to engage students. Lessons of this model focused on motivating students not to use drugs, addressing their intentions to use substances, and assisting them in identifying substances and generating resistance against pressure to use substances.

Project SUCCESS (Schools Using Coordinated Community Efforts to Strengthen Students) is a selective/indicated programme for high-risk students in alternative high schools, grounded in the Theory of Reasoned Action and Cognitive Behaviour Theory [98]. Master’s-level counsellors placed in schools deliver a 6–8 session Prevention Education Series to all students, then individually screen students for substance use and refer those needing further support to individual counselling, one of ten types of small groups, or community-based treatment; counsellors also conduct outreach to parents and the community.

Project Northland Chicago (PNC) is an adapted multi-component alcohol use preventive intervention designed specifically for urban, low-income, and multi-ethnic youth populations [99]. This intervention was designed to be implemented consecutively from 6th to 8th grade, with each year involving school, family, and community components. Major adaptations for urban youth included surface changes to curricula, expanded home programmes, peer-led community service projects rather than social activities, and greater emphasis on community organising with organisers focused in neighbourhoods.

Project Towards No Drug Abuse (TND) intervention strategy is used in three studies included in this scoping review [100,101,102]. It is a 12-session school-based substance abuse prevention programme designed for older, at-risk teens and emerging adults. The TND programme comprises 12 classroom sessions, each approximately 45 min in duration. It is delivered by trained health educators in selected classrooms over four weeks. The programme also includes a Motivational Interviewing (MI) booster component consisting of up to three sessions lasting approximately 20 min each.

The SAFEChildren intervention supports and smooths developmental management, social support, and school-family association for first-graders from families living in inner-city neighbourhoods [103]. Its major components are parent group sessions, child tutoring programme and booster intervention after two years of initiation of intervention.

Based on the social development and learning theory, the Seattle Social Development Project (SSDP) is an expanded version of Hirschi’s social control [104]. It assumes that delinquent behaviour can be avoided if children have strong social bonds with their close ones, such as their family and school. This framework was designed to prevent school failure, drug abuse and delinquency among low-income urban children. Intervention included three integrated components: classroom intervention, through trained teachers; child intervention, through social skills training for children; and parent intervention, through parent training sessions.

Another intervention guided by the Social Development Model is Raising Healthy Children (RHC). This intervention strategy aims to promote prosocial behaviour, thereby preventing antisocial behaviour [105]. It is a comprehensive multicomponent programme designed to promote healthy development in middle childhood by increasing prosocial behaviour through teachers’ training, students’ social and emotional skill sessions, and parents’ workshops.

The “Unplugged” programme is a 12-session school-based prevention curriculum designed to address alcohol, tobacco, and illicit drug use among adolescents aged 12–14 years [106]. The intervention is based on the Comprehensive Social Influence model and delivers interactive, evidence-based content through three integrated instructional blocks: the first block focuses on knowledge about substance use; the second block emphasises social skills training including effective communication, interpersonal relationships, self-awareness, and normative education to correct misperceptions about substance use prevalence; and the third block strengthens intrapersonal skills through practice in refusal, assertiveness, critical thinking, coping strategies, goal setting, decision-making, and problem-solving.

The Refuse, Remove, Reason (RRR) model employs Social Learning Theory and the mutual aid model to deliver the highest impact in the fewest sessions [107]. It is a brief, universal classroom-based substance abuse prevention programme designed for high school students to reduce alcohol use and change social norms around drug use. The program consists of: core classroom sessions, providing information about substances, their potential harms and refusal skills, including video segments; four homework assignments, including web-based videos and written self-reflection activities; and print material for increasing parents’ awareness of the program.

Ho’ouna Pono is another video-enhanced curriculum designed as a culturally grounded substance abuse prevention programme [108]. The core component is the brief video vignettes of Hawaiian youth engaged in realistic drug-related problem situations. The culture wall activity involved the discussion and application of Hawaiʻi Island cultural concepts to drug prevention. For example, the concept of puʻuhonua (place of refuge) is used to introduce the concept of psychosocial “protection” in lesson 2 of the pilot curriculum. Other lessons encourage youth to consider the applicability of Hawaiian cultural constructs (e.g., “ohana”, “aloha”) to drug prevention through the culture wall activity and facilitated discussions.

Finally, the Rehearsal Plus (R+) intervention is based on both cognitive and behavioural conceptualisations, using techniques to inculcate specific skills, enhance knowledge through cognitive procedures and to teach behavioural responses to augment functioning [109]. It is a training programme designed for third-grade students that emphasises skills development through multiple integrated components. Key components include: drug identification sessions; assertiveness training; decision-making training; and an eight-step sequence behavioural session This programme also includes elaborative rehearsal sessions to enhance students’ understanding and appreciation for desired responding through deeper processing of information.

### 3.2. Synthesis of Findings

#### 3.2.1. Theoretical Grounding and Outcomes

The review includes both theorised and atheorised models, enabling the empirical comparison between the two. An explicit theoretical foundation in intervention was found to be associated with more robust outcomes. For instance, Models grounded in Social Cognitive Theory [70,82,90,91] consistently improved targeted cognitive and behavioural outcomes, including reduced risk of substance use and enhanced refusal self-efficacy. Similarly, the Social Development Model [104,105] demonstrated sustained prosocial skill gains over multi-year follow-up periods. Furthermore, EU-Dap “Unplugged” [106] explicitly built on the Comprehensive Social Influence Model and reduced alcohol-related problem behaviours across seven countries; Project SUCCESS [98], grounded in the Theory of Reasoned Action and Cognitive Behaviour Theory, increased perceived harm from alcohol and marijuana; and CATCH My Breath [90,91] and Click City [95], both explicitly theory-driven (Social Cognitive Theory and the Prototype/Willingness Model respectively), produced significant reductions in e-cigarette initiation and vaping intentions. OurFutures Vaping [92], explicitly grounded in a comprehensive social influence and harm-minimisation approach, similarly produced a robust behavioural effect. Conversely, thirteen [67,68,73,74,75,77,78,79,80,92,93,100,101,102,103] out of sixteen models without theoretical underpinnings showed fluctuating outcomes (see Table 3).

**Table 3:** Intervention strategy of the school-based models.

Study ID	Name of Intervention	Country	Underlying Theories	Approach	Participants	Composition of Intervention	Skills Targeted
[77]	Across Ages’	U.S.A.	NS ^1^	P&P ^2^	School administration, students, parents, community, seniors	Senior-mentoring; community service; youth development curriculum; parent workshop	Self-esteem; coping; substance knowledge
[74]	ALF (Allgemeine Lebenskompetenzen und Fertigkeiten)	Germany	NS ^1^	P&P ^2^	School administration, students	12 teacher-led sessions (8 life skills + 4 substance-focused)	Communication; coping; critical thinking
[78]	All Stars	U.S.A.	NS ^1^	P&P ^2^	School administration, students, peers, parents	13 classroom sessions + peer-leader small groups; Symbolic rewards	Social binding; norm-setting; commitment
[80]	CAP (CLIMATE & Preventure)	Australia	NS ^1^	P&P ^2^	School administration, students, experts	Climate Schools: For all students; Twelve classroom lessons: alcohol & cannabis (20 min. online cartoon component + 20 min. group activity) Preventure: Only for students at high risk; Two group session delivered by registered clinical psychologist	Social–interpersonal, cognitive, and autonomy/emotion-management life skills; bonding; inclusion
[90,91]	CATCH My Breath	U.S.A.	Social Cognitive Theory	P&P ^2^	School administration, students, experts	4 classroom sessions; peer-led (small groups) activities	Refusal, resilience, critical thinking
[95]	Click City	U.S.A.	Theory of Planned Behaviour	P&P ^2^	School administration, students	4 interactive classroom modules (25 min); peer-leader facilitation; posters; PE companion activities	E-cigarette knowledge; refusal; identification of social/environmental influences
[79]	CLIMATE Schools	Australia	NS ^1^	P&P ^2^	School administration, students, parents	Classroom lessons including 15–20 min computer-based lesson & class activities	Cannabis/alcohol-related knowledge, harm minimization information, personality-specific coping strategies
[82]	Coping Power Program	U.S.A.	Social Cognitive; Social Learning; & Developmental Theory	P&P ^2^	School administration, students, parents, experts	33 boys’ group sessions + 16 parent sessions (15 months)	Social–cognitive processes; social problem-solving; pressure handling; parenting skills
[81]	Creciendo Juntos	Mexico	Health Promotion Theory; Sociology of Modernity, Positive Youth Development	P&P ^2^	School administration, students, parents, counsellors	400+ activity compendium + tailored group-based activities; monthly meeting; observational visits	Bonding, communication, conflict resolution, critical thinking, decision-making, competence development
[75]	D.A.R.E.	U.S.A.	NS ^1^	P&P ^2^	School administration, students, experts	Sessions (1 hr/week) by uniformed police officers Activities: Role play, homework assignment.	Pressure resistance, decision-making, self-esteem, critical thinking
[72]	D.A.R.E.’s EkiR	U.S.A.	Social Emotional Learning Theory	P&P ^2^	School administration, students, peers, experts	10 D.A.R.E. officer-led lessons; 5 videos; comic book	Decision-making; self-management; relationship and communication skills
[83]	DEVS (Drug Education in Victorian Schools Program)	Australia	Social Learning; Post-Structural Subjectivity; & Cognitive dissonance Theory	P&P ^2^	School administration, students, parents	18 lessons over 2 years; teacher training; workbooks	Drug knowledge; communication; critical disposition
[85]	DST (Drug Story Theatre)	U.S.A.	Strategic Prevention Framework (SPF)	P&P ^2^	School administration, students, peers	Live theatre performance + talk-back sessions	For students: Risk perception and prevention awareness For performers: Recovery and personal development skills
[86]	Fresh Start	Netherlands	Health Belief Model; Theory of Planned Behaviour; Transtheoretical Model; Social-Cognitive Theory.	P&P ^2^	School administration, students, parents	4 weekly lessons; peer pressure video; parents’ conference	Attitude towards smoking, alcohol use and cannabis use
[93]	HeadOn (Computer-based intervention)	U.S.A.	NS ^1^	P&P ^2^	School administration, students	Computer-based module [10]; “Skill Challenge” card games	Drug refusal & resistance; social skills; self-management skills
[89]	Healthy School & Drugs Project	Netherlands	Theory of Planned Behaviour; Social-Cognitive Theory.	P&P ^2^	School administration, students	Educational lessons over 3 years; school regulations on drug use; parental involvement; early detection & support system	Refusal skills; decision-making skills; self-esteem development
[108]	Ho’ouna Pono	U.S.A.	Social Influence Model	P&P ^2^	School administration, students, community members	Nine video-based lessons; interactive activities	Resistance & refusal
[67]	kiR REAL Group Model	U.S.A.	NS ^1^	P&P ^2^	School administration, students, peers, experts	10 classroom lessons; 5 videos; mutual-aid group component	Resistance; critical thinking
[73]	kiR ^3^	U.S.A.	NS ^1^	P&P ^2^	School administration, students	10 classroom lessons; 5 videos; booster activities; media campaign	Decision-making; resistance; risk assessment
[68]	kiR ^3^ (acculturation version)	U.S.A.	NS ^1^	P&P ^2^	School administration, students	10 classroom lessons; 5 videos; Latino/non-Latino/multicultural versions	Acculturation-linked; refusal confidence
[69]	kiR ^3^ (kiR-plus & kiR-AE)	U.S.A.	Communication Competence Theory	P&P ^2^	School administration, students	10 classroom lessons + 2 new lessons added in each version (kiR-Plus & kiR AE ^4^)	Resistance; anti-drug expectancies; refusal self-efficacy
[70]	kiR ^3^ (Entertainment–Education Videos	U.S.A.	Social Cognitive Theory; Narrative Engagement Theory	P&P ^2^	School administration, students	“urban” & “rural” video versions of original kiR	Drug offers resistance; refusal self-efficacy
[71]	kiR MREAL (Mantente REAL)	Mexico	Ecological Risk and Resiliency Theory; Communication Competence Theory; Narrative Theory.	P&P ^2^	School administration, students	2-day teacher training; 12 weekly lessons	Drug resistance; non-permissive substance use norms and attitudes
[92]	OurFutures Vaping	Australia	NS ^1^	P&P ^2^	School administration, students, experts	Web-based cartoon lessons; quizzes, class discussion or role plays	Refusal, decision-making, social competence, critical thinking
[96,97]	Project ALERT	U.S.A.	NS ^1^	P&P ^2^	School administration, students, experts	10 classroom sessions; class discussions; small group activities, role-playing exercises.	Drug identification; resistance
[98]	Project SUCCESS	U.S.A.	Theory of Reasoned Action, Social Learning Theory, Cognitive Behaviour Theory	P&P ^2^	School administration, students.	11 classroom lessons (6th grade) + 3 booster lessons (7th grade)	Resistance, self-efficacy, refusal
[99]	Project Northland Chicago (PNC)	U.S.A.	Theory of Triadic Influence; Perry’s planning model for adolescent health promotion	P&P ^2^	School administration, students, parents, community	6–10 peer-led classroom sessions per year; 4 home-based sessions per year Planned community service projects	Resistance, Self-efficacy, parenting
[87]	PULSAR	U.S.A.	NS ^1^	P&P ^2^	School administration, students, peers, experts	“At-risk” student identification; two-day retreat; post-retreat interviews	Communication and interpersonal; leadership and social competence; problem-solving skills; resistance and refusal skills; conflict resolution skills
[109]	R+ (Rehearsal Plus)	U.S.A.	NS ^1^	P&P ^2^	School administration, students, experts	Drug knowledge + assertiveness + behavioural + rehearsal Components	Assertiveness; decision-making
[105]	RHC (Raising Healthy Children)	U.S.A.	Social Development Model	P&P ^2^	School administration, students, parents	Teacher + child + parents training	Social, emotional & cognitive
[107]	RRR (Refuse, Remove, Reasons)	NS ^1^	Social Learning Theory; Mutual Aid Model	P&P ^2^	School administration, students, peers, experts	5-week curriculum; videos; homework	Risk perceptions
[84]	SHAHRP (School Health & Alcohol Harm Reduction Project)	Australia	Harm Reduction Theory	P&P ^2^	School administration, students, experts	Phase 1: Eight activity-based lessons Phase 2: Five booster lessons Includes student workbooks and videos	High-risk identification, alcohol-related management and planning
[104]	S.S.D.P. (Seattle Social Development Project)	U.S.A.	Social Control Theory; Social Development Model	P&P ^2^	School administration, students, peers, parents	Teacher + child + parents training	Social/family bonding; refusal skills
[103]	SAFEChildren	U.S.A.	NS ^1^	P&P ^2^	School administration, students	16-session tutoring; parent workshops; booster family sessions	Reading; interaction; parental
[88]	SUPEP (Substance Use Prevention Education Program)	Nigeria	HBM (Health Belief Model)	P&P ^2^	School administration, students	Teacher-delivered 12 weekly sessions + Q&A	Self-control and self-efficacy; drug resistance and resilience
[100,101,102]	TND (Project Towards No Drug Abuse)	U.S.A.	NS ^1^	Preventive	School administration, students, experts	12-session classroom curriculum; MI booster sessions (in-person/telephone)	Decision-making; motivation and behaviour change.
[94]	TGFD (Too Good for Drugs)	U.S.A.	Social Development Model; Social Learning; Social and Emotional Learning	P&P ^2^	School administration, students, parents, experts	10 weekly sessions; workbooks; homework	Goal setting; decision-making; interaction & bonding; self-management skills; drug resistance skills
[76]	TCYL (Take Charge of Your Life)	U.S.A.	Constructive Learning Theory	Preventive	School administration, students, experts	Lessons on substances and skills (specific for 7th and 9th grade level)	Communication, decision-making, assertiveness, refusal
[106]	EU-Dap “Unplugged”	7-EU Nations	Comprehensive Social Influence Model	Preventive	School administration, students.	12 classroom sessions	Resistance, peer pressure management, alcohol knowledge, decision-making

^1^ NS = not specified; ^2^ P&P = Preventive and Protective; ^3^ kiR= Keeping it R.E.A.L. (Refusal, Explain Avoid Leave); ^4^ PULSAR = Police, Public educators, and Peers Utilising Leadership skills of Students At Risk/As Resource.

Notably, Project Northland Chicago [99], explicitly built on the Theory of Triadic Influence and Perry’s planning model, showed no significant intervention effect on alcohol, tobacco or marijuana use despite high-fidelity implementation.

#### 3.2.2. Multi-Stakeholder Involvement and Intervention Effectiveness

The inclusion of multiple stakeholders consistently yielded broader and more durable outcomes than those confined to students and school administration alone. As mapped in Table 3, models like Across ages [77], SSDP [104], Healthy school and Drugs Project [89], Creciendo Juntos [81], Ho’ouna Pono [108] and RHC [105] that integrated teacher training, parental workshops and community participation showed significant improvements across academic performance, prosocial bonding, and reduced substance. The Across ages model also involved senior citizens (55+) as intergenerational mentors to improve students’ sense of self-worth [77].

Conversely, school-only models like HeadOn [93] and SUPEP [88] improved knowledge and attitudes without corresponding behavioural change. This pattern was not, however, universal. Project Northland Chicago [99] represented the fullest multi-stakeholder design in this review, yet the intervention as a whole produced no significant effect on alcohol or drug use relative to control. Similarly, CLIMATE Schools [79] and CAP [80] showed no significant reduction in alcohol or cannabis use expectancies.

#### 3.2.3. Cultural Adaptation as a Moderating Factor

Cultural tailoring significantly moderated intervention effectiveness, notably in the kiR programme family. The original kiR model, for seventh-graders using an ecological approach [67,69], produced strong baseline drug-refusal and decision-making skills. Culturally grounded adaptations for Mexican descent students produced more favourable substance use attitudes and behaviours, particularly among English-dominant high-risk students [68]. Cross-national transfer to a Mexican border city [71] similarly demonstrated reduced alcohol and drug use frequency, particularly marijuana use among males. An entertainment-education video adaptation tailored to urban and rural cultural contexts increased refusal self-efficacy and decreased future alcohol use [70]. However, the kiR Acculturation Enhancement version [69] increased substance use expectancies by 8th grade (Table 4).

The Hoʻouna Pono curriculum extended this pattern of cultural moderation to a Native Hawaiian population, embedding culturally grounded concepts such as puʻuhonua (place of refuge) and family-specific drug-offer scenarios within its resistance-skills lessons; the curriculum produced small but significant reductions in cigarette/e-cigarette and hard drug use for some cohorts, but not for alcohol or marijuana use [108]. Likewise, Creciendo Juntos was adapted to the sociocultural context of Mexican school communities and produced significant decreases in alcohol use, and among girls, in tobacco and marijuana use as well [81], further supporting cultural adaptation as a moderator of intervention effectiveness.

**Table 4:** Summary of the studies included in the scoping review.

Study ID	Name of Intervention	Covered Substances	Study Design				Population		Significant Outcome
			Method	N	Design	Duration	Age	Grade	
[77]	Across Ages’	ATOD ^5^	Mixed Method	729 (Pretest); 562 (Post-test)	Pretest–post-test control group design	3 years	NR ^2^	6th	Positive change in attitude towards school, their future, and older people; Reduced absenteeism; Improved knowledge about substance abuse; Increased sense of self-worth and feelings of well-being.
[74]	ALF (Allgemeine Lebenskompetenzen und Fertigkeiten)	Alcohol & Tobacco	Quantitative	256 IG ^3^ 192 CG ^4^	Quasi-experimental prevention study	1 year	10–11	5th	Decrease in nicotine use; Increase in distant attitude towards alcohol and nicotine use; Increase knowledge about life skills.
[78]	All Stars	ATOD ^5^	Mixed Method	Enrolled—102; Completed—96	Quasi-experimental study	4–5 months	NR ^2^	7th	Improved outcomes on all four skills targeted; Improved satisfaction levels; Improved parent involvement and teacher interaction.
[80]	CAP (CLIMATE & Preventure)	Cannabis, Alcohol	Quantitative	1712	4-arm cluster RCT ^1^	3 years	13–14	8th	Both Climate and CAP groups showed significantly greater cannabis-knowledge gains than control at 6, 12 and 24 months, converging by 36 months; No significant difference between Climate and CAP on knowledge; No significant difference in cannabis uses or cannabis-related harms between groups at any follow-up.
[90]	CATCH My Breath	Vaping	Quantitative	Enrolled—74 Follow-up—69	Cross-sectional Study	5 months	NR ^2^	6th–8th	Improved knowledge and attitude regarding e-cigarette use; No significant change in susceptibility of using e-cigarettes.
[91]	CATCH My Breath	Vaping, cigarette	Quantitative	12 schools 6 IG ^3^ 6 CG ^4^	Quasi-experimental	16 months	NR ^2^	6th–7th	Lower increase in ever e-cigarette use; Greater improvement in vaping knowledge and perceived positive outcome; Lower past-30-day combustible tobacco use.
[95]	Click City: Tobacco	E-cigarette, Cigarette	Quantitative	2673 (1327 IG ^3^ 1346 CG ^4^)	Cluster RCT ^1^	1 week	NR ^2^	5th	Decreased intentions and willingness to smoke and vape; Greater impact among high-risk students.
[79]	CLIMATE Schools	Alcohol	Mixed Method	764 397 IG ^3^ 367 CG ^4^	Cluster RCT ^1^	6 months	13–14	8th	Significantly increased alcohol knowledge at immediate and 6-month follow-up; Significantly reduced average weekly alcohol consumption immediately post-intervention, not sustained at 6 months; No significant difference between groups in alcohol expectancies, frequency of drinking to excess, or alcohol-related harm.
[82]	Coping Power Program	ATOD ^5^	Quantitative	60 (Children Group); 60 (Child & Parent Group) 63 (CG ^4^)	Pre–Post Comparison Group Design	2 years	NR ^2^	4th–7th (Boys)	Lower self-reported delinquent behaviour; Lower parent-reported substance use; Higher teacher-reported behavioural improvement at school.
[81]	Creciendo Juntos	Alcohol, Tobacco, Marijuana	Mixed Method	285	Mixed-methods trend study with epidemiological comparison data	4 years	NR ^2^	9th–10th	Significant decrease in lifetime alcohol use; Significant decrease in tobacco use among girls (lifetime and past-year); Significant reduction in past-month alcohol use among girls; Marijuana use decreased among girls but increased (non-significantly) among boys.
[75]	D.A.R.E.	Alcohol, Tobacco, Marijuana	Quantitative	2071	RCT ^1^	5 years	11–16	6th–10th	Short-term impact on positive drug attitudes, peer pressure resistance skills and improved perception of peer drug use; No significant impact on cigarette, alcohol or marijuana use in long-term follow-up.
[72]	D.A.R.E.’s EkiR	Cigarette	Quantitative	359 IG ^3^ 584 CG ^4^	Quasi-experimental pretest–post-test matched group design	NR ^2^	NR ^2^	6th	Increased definitional knowledge of the concepts included in the curriculum; Increased decision-making skills (including drug abuse knowledge and drug refusal skills); Increased confidence in reasoning for the refusal of cigarette offers; Increased resistance skills against peer pressure; Increased positive attitude towards police officers.
[83]	DEVS (Drug Education in Victorian School Program)	ATOD ^5^	Quantitative	1161 IG ^3^ 585 CG ^4^	Cluster RCT ^1^	2 years	13–16	8th–10th	Increased knowledge of alcohol and drug use; Improved communication skills, especially with parents; Reduction in alcohol consumption among drinkers; no change in drinking prevalence.
[85]	DST (Drug Story Theatre)	ATOD ^5^	Mixed Method	871	Pre–post survey design & focus interviews	2 years	NR ^2^	6th–11th	Improved knowledge about substance use disorder; Improved risk perceptions; Recovery benefits for performers.
[86]	Fresh Start	Alcohol, Cigarette, Cannabis	Quantitative	478 IG ^3^ 548 CG ^4^	Cluster RCT ^1^	10 months	12–13	NR ^2^	Attitudes toward smoking and cannabis use became even more negative; Attitudes toward alcohol use remained stable.
[93]	HeadOn (Computer-based intervention)	ATOD ^5^	Quantitative	113 (HeadOn group); 159 (Life-skill group)	School-based controlled trial	1 year	12–13	7th	Improved objective knowledge about drug abuse prevention; Positive outcomes regarding behaviour, intentions, attitudes, and beliefs against substance use, despite having higher risk factors at baseline.
[89]	Healthy School & Drugs Project	Alcohol, Tobacco, Marijuana	Quantitative	1156 students—IG ^3^ 774 students—CG ^4^	Quasi-experimental design	3 years	12–18	NR ^2^	Increased knowledge about substance use; Decreased tobacco and alcohol use; Increased the frequency of marijuana use; Limited effect on attitudes towards alcohol use and self-efficacy.
[108]	Ho’ouna Pono	ATOD	Quantitative	486	Dynamic wait-listed control group design	2 years	11–14	6th–8th	Flatter alcohol use growth trajectory only for the latest-exposed cohort, contrary to the hypothesised effect for earlier cohorts; Small but significant deceleration in cigarette/e-cigarette use growth trajectory for one cohort; significant deceleration in hard-drug use growth for one cohort; No significant effect on marijuana use; models for crystal methamphetamine use did not converge due to very low use rates.
[67]	kiR REAL Group Model	ATOD ^5^	Quantitative	Total sample—421; 115 IG ^3^; 306 CG ^4^	Longitudinal randomised control trial	6–7 months	10–11	5th	No significant behavioural change; No significant differences in usage of alcohol, cigarette, or inhalant; REAL Group participants were not significantly different from non-participants in vulnerability levels.
[73]	kiR ^3^	Alcohol, Cigarette, Marijuana	Quantitative	4622	RCT ^1^	2 years	NR ^2^	7th	No gender difference in recent substance use; the anti-drug norm was more effective in fostering boys than girls; Effective among more acculturated Latino adolescents, who were at higher risk of initiating or increasing substance use than less acculturated adolescents.
[68]	kiR ^3^ (acculturation version)	Alcohol, Cigarette, Marijuana	Quantitative	2146	Pretest–post-test experimental design	14 months	NR ^2^	7th–8th	Students of Mexican descent showed more favourable outcomes in substance use and attitudes towards drug use; English-language dominant students (high-risk group) responded more positively to the intervention
[69]	kiR ^3^ (kiR-plus & kiR-AE)	Alcohol, Cigarette, Marijuana	Quantitative	1984	RCT ^1^	4 years	NR ^2^	5th–8th	No positive impact—students who received kiR-Plus showed an increase in substance use and more positive substance use expectancies by 8th grade.
[70]	kiR ^3^ (Entertainment–Education Videos	Alcohol	Quantitative	1464 (501—urban students; 963—rural students)	RCT ^1^	1 year	13–14	7th	Increased refusal self-efficacy; Decrease in the likelihood of future alcohol use.
[71]	kiR MREAL (Mantente REAL)	ATOD ^5^	Quantitative	1418	Cluster RCT ^1^	7 months	NR ^2^	7th	Students of IG3 used alcohol and illicit drugs, except marijuana, less frequently; Males (IG3) reported desirable intervention effects for marijuana use.
[92]	OurFutures Vaping	Vaping	Quantitative	2449 IG ^3^ 2708 CG ^4^ Follow-up—4956	Cluster RCT ^1^	12 months	12–14	7th–8th	Reduced odds of using e-cigarettes; Improved knowledge about e-cigarette use.
[97]	Project ALERT	Alcohol, Cigarette, Marijuana	Quantitative	5782 (participated initially); 4940 (completed all three waves)	RCT ^1^	2 years	NR ^2^	6th–8th	Limited effect in an AYP (adequate yearly progress) school for cigarette and alcohol use; Negative effect on marijuana uses in AYP schools.
[96]	Project ALERT	Alcohol, cigarette, Marijuana	Quantitative	4276 2553 IG ^3^ 1723 CG ^4^	Cluster RCT ^1^	18 months	NR ^2^	7th–8th	Reduced cigarette initiation and current/regular smoking; Reduced marijuana initiation; Reduced alcohol misuse and high-risk; No significant effect on alcohol initiation or current use.
[98]	Project SUCCESS	ATOD	Quantitative	2249	Cluster RCT ^1^	2 years	14–18	9th 12th	Increased perceived harm from alcohol and marijuana use; Mixed effects on normative beliefs.
[99]	Project Northland Chicago (PNC)	ATOD	Quantitative	4259	Group RCT ^1^	3 years	11–14	6th–8th	No significant differences were found for any substance usage; Higher participation in the home-based parent programme associated with a significantly lower drug-use trajectory and a marginally lower alcohol use trajectory; Non-significant trend toward reduced ability of minors to purchase alcohol commercially in intervention communities.
[87]	PULSAR ^6^	Drugs & Alcohol	Qualitative	05	Experiential study design	6 weeks	NR ^2^	7th	Intervention facilitated self-reflection on attitude toward substance abuse and school; Students gained skills that addressed individual needs, fostered relationships with adults and peers; Increased protective factors and resilient characteristics.
[109]	R+ (Rehearsal Plus)	Alcohol, Tobacco, Marijuana	Mixed Method	14 students—R+ 12 students—general information 8 students—CG ^4^	Pretest–post-test control group	8 weeks	8–10	3rd	Significant improvement in drug knowledge in R+ & GI; Significant improvement in general knowledge/self-esteem in R+ & GI; Assertiveness and decision-making skills showed no significant difference; R+ outperformed the GI and control group in behavioural and rational skills.
[105]	RHC (Raising Healthy Children)	ATOD ^5^	Quantitative	156 (full intervention); 141 (parent training only); 220 (CG ^4^)	Non-randomised controlled trial	33 years	6–39	From 1st	Improved academic performance; Reduced problem behaviour (includes delinquency and drug use); Better mental health outcome for IG3 and their children in the follow-up, i.e., positive intergenerational effects.
[107]	RRR (Refuse, Remove, Reasons)	ATOD ^5^	Quantitative	678 IG ^3^; 674 CG ^4^	Quasi-experimental design	3 Months	15	NR ^2^	Reduced instances of getting drunk from alcohol; Decreased social norms and acceptance of alcohol and cigarettes; Increased perceptions of risk and negative consequences of drug use;
[84]	SHAHRP (School Health & Alcohol Harm Reduction Project)	Alcohol	Quantitative	2300	Quasi-experimental	32 months	12–17	NR ^2^	Reduced alcohol-related harm and risky drinking behaviour; Improved alcohol-related knowledge and attitudes.
[104]	S.S.D.P. (Seattle Social Development Project)	Alcohol, Tobacco, Marijuana	Quantitative	177 (initially) −102 CG ^4^; 75 IG ^3^ 106 (completed) −62 CG ^4^; 44 IG ^3^	Self-report survey and longitudinal study	6 years	NR ^2^	1st–6th	Increased classroom involvement among girls of the IG3; Improved social skills and study skills in the boys of IG3; Antisocial peer interactions were significantly reduced amongst the boys of the IG3; No significant difference reported in the norms of substance use & delinquency measure between the IG3 & CG4.
[103]	SAFEChildren	Alcohol, Tobacco, Marijuana	Mixed Method	424 (consenting families); 375 (completed)	RCT ^1^	13 years	5–18	K.G.-12th	No statistically significant long-term direct effects of intervention on most outcomes
[88]	SUPEP (Substance Use Prevention Education Program)	ATOD ^5^	Quantitative	200 (initially) 126 (completed): 75 (IG ^3^) & 51 (CG ^4^)	Quasi-experimental pre-post test design	5 months	14–20	NR ^2^	Increased knowledge of psychoactive substances; Improved substance use attitude; No significant reduction in substance use.
[100]	TND (Project Towards No Drug Abuse)	ATOD ^5^	Quantitative	1074	Randomised treatment-control design	1 year	14–19	9th–12th	Significantly reduced hard drug and alcohol use; No impact on cigarette and marijuana use.
[101]	TND (Project Towards No Drug Abuse)	ATOD ^5^	Quantitative	1186	RCT ^1^	1 year	14–18	9th–12th	Significantly reduced future ATOD usage; No impact on any factors of risky sexual behaviour.
[102]	TND (Project Towards No Drug Abuse)	ATOD ^5^	Quantitative	1867	RCT ^1^	5 years	14–19	9th–12th	Significantly reduced hard drug use, both in short and long term; No impact on cigarettes, alcohol or marijuana use.
[94]	TGFD (Too Good for Drugs)	ATOD ^5^	Quantitative	Pre-survey stage (10,762)	Stratified randomised treatment-control design	6 months	11–14	K-12th	Suppressive effect on high-risk students; Strengthening effect on Risk & Protective skills and attitudes of high-risk students. Diminished students smoking behaviour.
[76]	TCYL (Take Charge of Your Life)	Alcohol, Tobacco, Marijuana	Quantitative	17,320 (baseline); 10,434 (11th-grade completers)	Cluster RCT ^1^	5 years	NR ^2^	7th–11th	Iatrogenic effect—significantly higher 30-day alcohol and cigarette use among treatment students, concentrated among baseline nonusers and white students; No significant effect on marijuana use or getting drunk overall; Significant beneficial effect (reduced marijuana use) among students who were already using marijuana at baseline.
[106]	EU-Dap “Unplugged”	ATOD ^5^	Quantitative	5541	Cluster RCT ^1^	18 months	12–14	7th 8th	Significantly reduced the risk of alcohol-related behavioural problems.

^1^ RCT = randomised controlled trial; ^2^ NR = not reported; ^3^ IG = intervention group; ^4^ CG = control group; ^5^ ATOD = Alcohol, Tobacco, and Other Drugs; ^6^ PULSAR = Police, Public educators, and Peers Utilising Leadership skills of Students At Risk/As Resource. NOTE: PULSAR model (N = 5) is a qualitative experimental study. Findings are not statistically generalizable.

#### 3.2.4. Dosage, Duration and Developmental Targeting

Interventions varied in duration and intensity, but patterns emerged. Brief models like the original kiR [73], RRR [107], and Fresh Start [86] produced short-term attitudinal and skill gains, while multi-year interventions like DEVS [83], Project ALERT [96,97], Creciendo Juntos [81], SHAHRP [84] and SSDP [104] were better positioned to observe behavioural changes and sustained protective factors. Early-grades programmes built skills; middle-school programmes addressed peer pressure and beliefs [89,104] (Table 4). Long-term follow-up data from Project TND sharpens this picture. Delivered in only nine sessions, it nonetheless produced a hard-drug-use reduction that was still detectable up to five years later among continuation high school students [102]. PNC [99] and TCYL [76] sit at the opposite end of the dosage spectrum, multi-year booster design, yet still produced no significant positive effect on alcohol or drug use, reinforcing that duration and session count cannot substitute for content-population fit.

Moreover, the CAP study [80] offers a particularly clear illustration of dosage decay over a longer observation window: both the universal Climate and combined CAP groups showed significantly greater cannabis-knowledge gains than control at 6, 12 and 24 months, but this advantage had disappeared by the 36-month follow-up as the control group’s knowledge caught up.

#### 3.2.5. Null, Partial and Iatrogenic Findings

The recognition of interventions that produced null, mixed, or unintended negative outcomes is critical for evidence-informed programme design. The REAL Group Model [67], an extension of the kiR program, showed no significant group differences in substance use, cultural pride, or self-esteem, and intervention group participants showed greater increases in marijuana use at post-test. Similarly, SAFEChildren [103] reported no statistically significant long-term direct effects on most outcomes after a 13-year follow-up, despite a robust RCT design. Project ALERT [96] demonstrated limited effectiveness in schools (AYP) and produced iatrogenic effects and increased substance use in non-AYP schools compared to control. SUPEP [88] improved knowledge and attitudes but produced no significant behavioural reduction in substance use. The kiR-Plus version [69] was associated with higher substance use expectancies by 8th grade.

Furthermore, EU-Dap “Unplugged” showed a related partial pattern: it reduced alcohol-related problem behaviours but did not reduce the overall frequency of alcohol consumption [106], while Project SUCCESS increased perceived harm from alcohol and marijuana yet saw peer support and normative beliefs about alcohol move in an unfavourable direction relative to controls [98] illustrating that a single curriculum can simultaneously succeed and fail across the different mechanisms it targets.

The TCYL trial provides a further, larger-scale example of iatrogenic risk. Delivered as a universal, non-targeted curriculum across 83 school clusters, TCYL produced significantly higher 30-day alcohol and cigarette use among treatment students by the 11th grade, an effect concentrated among students with no substance use experience at baseline and among white students [76]. Hoʻouna Pono similarly produced a partial and counterintuitive pattern, a finding the original authors attributed to unmeasured baseline risk factors rather than a true iatrogenic effect [108].

### 3.3. Key Outcome

Effectiveness of forty-three school-based substance use models was measured using: i. randomised controlled trial (twenty-three studies), including clustered and group RCT; ii. quasi-experimental study design (eight studies); iii. pretest–post-test design (five studies); iv. non-randomised controlled trial (1 study); v. school-based controlled trial (one study); vi. self-report survey and longitudinal study (one study); vii. cross-sectional study (one study); viii. mixed-methods trend study (one study); ix. dynamic wait-listed control group design (one study); and x. experiential (qualitative) study design (one study). Classes ranged from kindergarten to 12th grade (refer to Table 4). Findings from small studies were interpreted cautiously, not equated with multisite RCTs.

These studies report impacts ranging from cognitive and attitudinal changes to psychosocial and behavioural skills essential for preventing children from initiating substance use. Studies examining intervention models, such as Across Ages, the Healthy School and Drug Project, R+, SUPEP, DST, All Stars, CATCH My Breath, Project SUCCESS. and HeadOn, confirmed knowledge-related outcomes [77,78,85,88,89,90,91,93,95,98,109].

Correspondingly, certain intervention models such as HeadOn, ALF, PULSAR, RRR, Click City and SUPEP successfully developed or retained the negative attitudes of the participants against substance use [74,87,88,93,107]. However, the study including Fresh Start model highlighted that the intervention does not have any significant impact in fostering negative attitudes among school students towards substance use [86].

Intervention strategies employed in some models, including ALF, Coping Power Program, RHC, Healthy School and Drug Project, and SUPEP, have led to a significant reduction in substance use among students at risk [74,82,88,89,105]. The revised Project ALERT [96], CATCH My Breath [90,91], and EU-Dap “Unplugged” [106] produced comparable behavioural reductions, curbing cigarette and marijuana initiation, e-cigarette uptake, and alcohol-related problem behaviours respectively, and OurFutures Vaping [92] produced a comparable reduction in the odds of past 12-month e-cigarette use. Similarly, the RRR intervention not only led to a considerable decrease in substance use but also resulted in a significant reduction in drinking [107].

Similarly, the Creciendo Juntos achieved a significant reduction in lifetime alcohol use as well as significant reductions in tobacco and alcohol use among female students specifically [81], reinforcing that sustained, multi-stakeholder models can achieve measurable reductions in substance use among school populations.

By contrast, the CLIMATE Schools and the CAP study demonstrate that knowledge gains do not automatically translate into sustained behavioural change: CLIMATE produced strong, durable knowledge gains but only a short-lived reduction in alcohol consumption [79], while CAP produced durable cannabis-knowledge gains without a detectable reduction in cannabis use or harms [80], underscoring that knowledge and behavioural outcomes should be evaluated as distinct dimensions of programme success.

Studies exploring the kiR model, in its original or modified form, have highlighted its profound effectiveness in increasing knowledge regarding substance use. This model not only enhances the drug-refusal skills of the participant and their resistance skills against peer pressure but also significantly improves their decision-making skills and decreases the likelihood of future alcohol use [68,70,71,72,73]. Notably, two studies based on a modified version of the kiR model [67,69] diverged from these positive outcomes, showing no significant difference between children who received the intervention and those in the control group (Refer to Table 4). The kiR intervention has had a notable impact on indigenous students through cultural adaptation that resonates with their unique values and experiences [68,73].

Apart from direct results on substance use and knowledge and attitude towards it, the outcome of reviewed studies has also provided significant impacts of intervention models on various skills that indirectly facilitate building a healthy life free from substances. These skills include self-awareness [78], self-efficacy, self-worth [70,77], life skills [74], problem-solving skills [78], behavioural skills [82], resilience skills [87], resistance and refusal skills [70,72,77], communication skills [72,78], decision-making skills [72,109], critical thinking skills, and relationship and bonding skills [78].

### 3.4. Critical Appraisal of Included Studies

Methodological quality, appraised using the MMAT, ranged from moderate to high and varied systematically by design. The 23 RCTs/cluster RCTs showed the strongest internal validity, though allocation concealment and blinding were inconsistently reported. Quasi-experimental and pre–post studies were of moderate quality, limited by non-randomisation and sparse attrition/confounding reporting. Remaining quantitative and mixed-methods studies generally met applicable criteria, with self-reported outcomes a recurring limitation. The single qualitative study (PULSAR, *n* = 5) showed strong coherence but limited generalisability. No study was excluded on quality grounds, but weaker studies were interpreted cautiously throughout.

## 4. Discussion

This review covers 43 studies including 33 intervention models, in original or modified version, for substance use among children. A striking trend across the studies is that the original curricula are iteratively adapted rather than being replaced. The kiR appears in this review with several distinct adaptations, including EkiR, kiR-Plus, kiR-AE, REAL group model and MREAL, a culturally localised and translated version for children in Mexico [69,70,71,72,73]. Similarly, SAFEChildren demonstrates an adapted and extended family-focused model deployed with a younger population, featuring a multi-year booster structure [103]. However, the results of several adapted versions were not similar but rather worse than the originals, highlighting that each version must be examined as a separate intervention rather than assuming its inherent effectiveness from its base model.

More specifically, there were two culturally adapted versions of kiR: kiR AE [69], delivered to a younger population than the targeted population of the original version; and the MREAL intervention [71], which fully relocalised the language and curriculum of the original model for Mexican children, but the targeted grade (7th) was the same as the original. The kiR-AE model showed weaker and mixed results, whereas the MREAL produced more positive outcomes. This difference highlights a well-documented tension in the adaptation studies, suggesting that when adaptations drift too far from the validated core, it risks losing effectiveness even if they are culturally relevant [110]. This finding highlights that cultural adaptation of an intervention can be successfully implemented if its changes are made on the lines of cultural framing without alteration in the original target or theoretical core.

Another pattern recognised in this review is that the intervention founded in explicit behavioural theory generated more consistent and sustained outcomes than the atheorised model. This finding is consistent with a meta-analysis conducted in 1997 on the effectiveness of school-based drug prevention programmes [111]. This analysis revealed that prevention programmes based on social-influence theory were substantially more effective than non-interactive, knowledge-only programmes. The current review extends this finding and highlights that the effectiveness of an intervention lies in theoretical coherence. This can be because theories give a model a single mechanism to work upon, keeping its content, dosage and outcome measurement aligned. This can be clearly demonstrated through the contrast between SHAHRP and D.A.R.E.: SHAHRP’s explicit harm-minimisation rationale sustained reductions in alcohol-related harm well beyond programme completion [84], whereas D.A.R.E.’s broader, less theoretically anchored combination of cognitive, affective and social-skills content produced almost no significant behavioural effect [75], reinforcing that theoretical coherence, rather than breadth of content, underlies durable change.

Project SUCCESS illustrates just how far this multi-component logic can be pushed: placing master’s-level counsellors inside alternative high schools to combine classroom lessons with individual counselling, small groups, and outreach to parents and the community increased perceived harm from alcohol and marijuana, yet the same design saw peer support and normative beliefs about alcohol move in an unfavourable direction relative to controls, showing that adding stakeholders broadens a programme’s reach without guaranteeing that every mediator moves the same way [98]. By contrast, the teacher-only EU-Dap “Unplugged” trial, run across seven European countries without counsellors, parents or community partners, still reduced alcohol-related problem behaviours, indicating that classroom-only delivery is not inherently insufficient so much as differently suited to some outcomes (behavioural harms) than others (overall consumption, which it did not change) [106].

The models that included components like parents, community members, senior mentors, and peer performers rather than school curricula only showed significant behavioural changes. Meanwhile, the school-only programmes like HeadOn and SUPEP showed improvement only in knowledge and attitudes without demonstrating similar results in usage. This highlights that social factors like family, peer and community influences substance use among adolescents, which cannot be effectively addressed by classroom lessons alone. Therefore, effective prevention strategies need to incorporate family and community-linked components rather than intensifying classroom sessions in isolation.

The limited impact of kiR-Plus and no statistically significant long-term effects of SAFEChildren showed that duration, dosage and developmental intervention timings cannot be treated as independent design choices. The optimal approach requires delivering the right amount of content at the specific developmental stage and sustaining it long enough that the protective effects do not fade [111].

Furthermore, Project TND, only nine sessions long, produced a reduction in hard drug use that was still detectable up to five years later among continuation high school students [100,102], whereas the far more extensive 11-lesson-plus-booster Project ALERT [96,97] curriculum saw its sole short-term effect on alcohol use disappear within a single year of follow-up in a separate national trial. Session count alone, in other words, predicts durability poorly; what seems to matter more is whether the content and population are well matched, since Project ALERT’s revised, more intensive version, adding smoking-cessation and alcohol-focused lessons, did produce durable-looking short-term gains across three substances when tested with a different, Midwestern cohort.

Apart from developmental stage and dosage decay, grouping of high-risk adolescents for peer resistance strategies may inadvertently amplify the risky peer interaction rather than weakening harmful peer dynamics [112]. Similarly, a finding of Project ALERT proposed that students in low-performing schools may face greater challenges in applying the cognitive strategies. This highlights that contextual factors within school accounted for weaker outcomes rather than a flaw in the curriculum itself.

Interestingly, Creciendo Juntos/Madrid Tutor demonstrates that sustained, theory-driven, whole-school implementation, integrating teachers, families, and the broader school community into a permanent programme structure rather than a time-limited curriculum, can achieve significant reductions in alcohol use overall and in tobacco, alcohol, and marijuana use among female students specifically [81]. This finding lends further support to this review’s emphasis on multi-stakeholder involvement and dosage sustained over a developmentally appropriate period, and suggests that permanence and institutional embeddedness may be an additional design principle worth incorporating into future integrated, corrective models.

The CAP study adds a further dimension to this discussion by directly testing whether combining universal and selective prevention produces additive benefits. Adding the selective, personality-targeted Preventure programme to the universal Climate curriculum did not improve cannabis-related knowledge, use, or harm outcomes beyond the universal programme alone [80], suggesting that tiered universal-plus-selective designs are not automatically superior to universal delivery and that the added value of a selective layer may depend on how closely it targets the specific substance and risk mechanism in question. The CLIMATE Schools, evaluated as a cross-validation trial in a new set of schools, reinforces a further point raised throughout this review: computerised, standardised delivery can achieve consistent knowledge gains with minimal teacher training, but short lesson counts delivered over a single term appear insufficient to sustain behavioural change beyond the immediate post-intervention period [79]. Together, these findings suggest that future integrated, corrective models should consider not only whom they involve, but how precisely their selective or corrective component is matched to the substance and risk factor it targets, rather than assuming that adding layers of intervention will proportionally add to effectiveness.

The current review underscores the significance of including preventive and protective models in the school curriculum to enhance the students’ knowledge regarding substance use by consequently inculcating students with essential skills that increase their resistance against substance use, ultimately reducing the risk of substance use among adolescents. Unfortunately, the common approach adopted in school policies towards children using substances is deterrent and punitive. Substance use is considered misconduct by schools and is dealt with using a zero-tolerance approach. Students who use tobacco and alcohol in schools are subjected to disciplinary actions such as suspension, expulsion, or fines [113]. Such traditional punitive responses are detrimental, resulting in an increased risk of dropout, increased absenteeism, lower self-esteem, enhanced substance use, and the possibility of drug use among students [114]. This review chronicles the impact of preventive school-based interventions implemented by schools. Additionally, integrated models involving parents, senior citizens, community members, and experts with cultural adaptation significantly improved knowledge, attitude, and perception of substance use and its negative consequences. However, this does not provide an unconditional guarantee of success, as illustrated by the non-significant overall impact on behavioural outcomes of Project Northland Chicago, a multicomponent long-term intervention grounded on a theory. This intervention demonstrates that the potency of individual components, and how well each is matched to the population, may matter more than the other factors involved. As impactful interventions, these models guide future research on developing school-based corrective intervention models.

Only CATCH My Breath, Click City, and OurFutures Vaping were purpose-built, or substantially rebuilt, for e-cigarette prevention, and each had to invent new content because e-cigarettes carry a different harm profile, different flavour marketing, and different social norms than combustible products [90,91,92,95]. Given how rapidly youth vaping has risen, curriculum development in this field appears to be lagging behind the changing landscape of adolescent substance use, and future school-based programming may need the same kind of purpose-built approach for e-cigarettes that this review otherwise recommends for corrective, early-stage interventions.

Furthermore, a substantial finding highlighted by the reviewed literature is the absence of corrective intervention models, despite the documented requirement for therapeutic approaches for adolescents [115] engaging in the early stage of substance use. This highlights a crucial actionable gap for future programme development, with further alignment with the “sociology of acceptance” [116]. The review advocates the construction of alternative school-based corrective intervention models. Such a model can replicate the success of school-based intervention models in correcting substance users in school settings, especially where the student is consuming alcohol or tobacco and is in the initial stages, with no medical intervention required. The authors strongly recommend the development of such therapeutic interventions in school settings, which will decrease the stigma attached to substance use, reporting, and rehabilitation.

## 5. Conclusions

This scoping review demonstrates that school-based preventive interventions for adolescent substance use are most effective when several design elements align: explicit theoretical grounding, involvement of multiple stakeholders beyond the classroom, cultural adaptations that preserve developmental and theoretical fidelity while changing cultural framing, and dosage calibrated to the recipient’s developmental stage. Where these elements were absent or misaligned, as seen in the age-mismatched kiR-Plus and kiR-AE versions, the dosage-decay pattern in SAFEChildren, and the peer-grouping effect in the REAL Group Model, interventions produced null or even iatrogenic outcomes, indicating that harm in this literature is patterned and anticipatable rather than random.

Across nearly all models, knowledge and attitude change proved easier to achieve than behavioural change, underscoring the continued importance of active skill-building over information delivery. Interventions in school settings remain a uniquely valuable strategy because of their sustained access to large numbers of children and their existing infrastructure and workforce. However, this review also confirms a clear gap: no corrective intervention model exists for students already in the early stages of substance use, despite schools’ continued reliance on punitive, zero-tolerance responses.

Adding components does not guarantee stronger effects: Project SUCCESS, “Unplugged,” and the CAP study show that precision in matching content, dosage, and selective/corrective layers to population and risk mechanisms matters more than programme scale. Conversely, Creciendo Juntos/Madrid Tutor’s sustained, whole-school model suggests institutional permanence is a further design principle worth pursuing.

Building on the design principles identified here, the field’s next priority should be an integrated, theory-driven corrective model grounded in the sociology of acceptance, offering a non-stigmatising, therapeutic alternative for early-stage adolescent substance use.

## 6. Limitations

- This review includes literature published only in the English language, thereby excluding models which could be analysed in any other language.
- The majority of the relevant intervention models were applied from the US (30 of 43). This substantially limits the generalizability of findings to non-Western countries. Future reviews will be conducted as soon as relevant interventions are studied in low- and middle-income countries like Sub-Saharan Africa and Southeast Asia.
- The review must be interpreted cautiously as the heterogeneity in the study design and the iatrogenic and null findings in some models complicate direct comparisons of the models.
- This review’s protocol was not formally pre-registered; readers may wish to bear this in mind alongside the review’s other transparency measures.
- The search drew on two databases, SCOPUS and Web of Science; readers should note that the evidence base reflects this scope, alongside the English-language restriction already noted, rather than a broader multi-database or grey-literature search.
- Formal inter-rater reliability (κ = 0.75) is reported for title/abstract screening; full-text screening and data charting were carried out jointly by the review team, and readers may wish to keep this collaborative process in mind when weighing these stages of the review.

## Figures and Tables

**Table 1:** PCC framework.

Criteria	Determinants
(P) Population	School-Going Children
(C) Concept	Intervention models for substance use: Preventive, protective, and corrective
(C) Context	Curriculum (English only) across the world, including school administration, parents, community and peer groups.

**Table 2:** Search strategies.

Database	Search String	Limit	No. of Records
SCOPUS	(“Drug use” OR “Substance use” OR “Drug abuse” OR “Substance abuse” OR “Alcohol” OR “Vaping” OR “e-cigarette” OR “Cigarette”) AND (“Children” OR “School-going” OR “Adolescent” OR “Youth” OR “Students” OR “Young”) AND (“Education” OR “Prevention” OR “Protection” OR “Rehabilitation” OR “Counselling” OR “Integrated” OR “Corrective” OR “Evaluation”) AND (“Model” OR “Strategy” OR “Approach” OR “Intervention” OR “Framework” OR “Project” OR “RCT”)	English Only Journal Articles Published	19,261
Web of Science	(“Drug use” OR “Substance use” OR “Drug abuse” OR “Substance abuse” OR “Alcohol” OR “Vaping” OR “e-cigarette” OR “Cigarette”) AND (“Children” OR “School-going” OR “Adolescent” OR “Youth” OR “Students” OR “Young”) AND (“Education” OR “Prevention” OR “Protection” OR “Rehabilitation” OR “Counselling” OR “Integrated” OR “Corrective” OR “Evaluation”) AND (“Model” OR “Strategy” OR “Approach” OR “Intervention” OR “Framework” OR “Project” OR “RCT”)	English Only Journal Articles Published	574
Total			19,835

## Data Availability

No new data were created or analysed in this study. Data sharing is not applicable to this article.

## Supplementary Materials

The following supporting information can be downloaded at: https://www.mdpi.com/article/10.3390/ijerph23081073/s1. File S1: Preferred Reporting Items for Systematic reviews and Meta-Analyses extension for Scoping Reviews (PRISMA-ScR) Checklist. Reference [117] is cited in the Supplementary Materials.
